# Comparative Study of the Effects of Carvacrol and *p*-Cymene on the Motor Activity of Rats and Movement of *Caenorhabditis elegans*

**DOI:** 10.3390/molecules31071119

**Published:** 2026-03-28

**Authors:** Oliver Stošić, Dragana Medić, Djordje S. Marjanović, Tihomir Marić, Veljko Savić, Jelena Nedeljković Trailović, Nemanja Zdravković, Saša M. Trailović

**Affiliations:** 1 Department of Experimental Surgery and Laboratory Animal Breeding, Veterinary Service Center, Military Health Department, Ministry of Defense, 11000 Belgrade, Serbia; oliver.stosic@gmail.com (O.S.); vejkosavic65@gmail.com (V.S.); 2 Department of Pharmacology and Toxicology, Faculty of Veterinary Medicine, University of Belgrade, 11000 Belgrade, Serbia; dragana.medic@vet.bg.ac.rs (D.M.); marjanovicd@vet.bg.ac.rs (D.S.M.); tihomir.maric@vet.bg.ac.rs (T.M.); 3 Department of Animal Nutrition and Botany, Faculty of Veterinary Medicine, University of Belgrade, 11000 Belgrade, Serbia; tjelena@vet.bg.ac.rs; 4 Scientific Institute of Veterinary Medicine of Serbia, 11000 Belgrade, Serbia; nemanja.zdravkovic@nivs.rs

**Keywords:** carvacrol, *p*-cymene, rats, *Caenorhabditis elegans*

## Abstract

The active constituents of essential plant oils (EOAIs), monoterpenoid carvacrol and monoterpene *p*-cymene, are widely distributed in many aromatic plants and their products. They differ in that carvacrol has a phenolic functional group. The numerous pharmacological effects of these two EOAIs are well known. In different doses/concentrations, they exhibit analgesic, neuroprotective, vasorelaxant, anti-inflammatory, antiviral, antibacterial and antiparasitic effects. The acute toxicity of carvacrol and *p*-cymene in rats and the free-living nematode *Caenorhabditis elegans* was investigated. Furthermore, the impact of subacute administration of these two terpenes on general health, CNS integration, i.e., motor coordination and balance of rats, as well as their effects on the movement of adult *C. elegans*, was also examined. The aim was to compare the effects and describe in more detail the selective toxicity of carvacrol and *p*-cymene. The calculated LD_50_ value of carvacrol was 790.15 ± 1.15 mg/kg, while the LD_50_ value of *p*-cymene is above 3000 mg/kg. Tested doses of carvacrol and *p*-cymene administered for 28 days (50, 100, and 200 mg/kg) did not exert any effect on the CNS of rats or cause any clinical disorders. LC_50_ value of carvacrol for adult *C. elegans* was 184.13 ± 1.51 μM and for *p*-cymene 1268 ± 1.65 μM. In subacute testing, carvacrol showed negative effects on *C. elegans* reproduction, distance traveled, movement speed and rotational index at lower concentrations than *p*-cymene, indicating higher toxicity, which may be due to its phenolic structure. On the other hand, although less toxic to *C. elegans*, *p*-cymene exhibited a specific effect on worm motility, with more rolling which should be further investigated, and can be a consequence of cuticle damage or loss of orientation.

## 1. Introduction

Active ingredients of essential plant oils (EOAIs), monoterpenoid carvacrol and monoterpene *p*-cymene are increasingly being studied and used in various therapies [1,2,3]. Both are widely distributed in several aromatic plants and their products. *P*-cymene is a main compound in essential oil of genus *Artemisia* (Asteraceae), *Protium* (Burseraceae), *Origanum*, *Ocimum*, *Thymus*, (Lamiaceae), and *Eucalyptus* (Myrtaceae) [4,5], while carvacrol is the major natural constituent in the essential oil of aromatic plants belonging to the family *Lamiaceae*, such as oregano and thyme. Carvacrol is a derivative of cymene, it is formed during the biosynthesis process in plants, and it is fundamentally categorized as a monoterpenoid due to its phenolic functional group. Numerous pharmacological effects of these two EOAIs have been described. They are known to exhibit analgesic, neuroprotective, vasorelaxant, anti-inflammatory, antiviral, antibacterial, and antiparasitic effects [2,6,7].

A large number of studies confirm the specific pharmacological properties of the active ingredients of plant oils. For example, monoterpenes *p*-cymene, carvacrol and thymol in a dose of 20 mg/kg reduced lung emphysema and inflammation in mice with no significant differences, suggesting that the presence of hydroxyl group in the molecular structure of thymol and carvacrol do not play a central role in the antiinflammatory effects [8]. Oral doses of *p*-cymene with different pharmacodynamic effects in animals reported in the literature range from 20 to 100 mg/kg of body weight [9]. On the other hand, single doses of carvacrol that exert effects on the CNS range from 150 and 450 mg/kg [10], while doses that improved the functionality of endothelial progenitor cells, contributing to the reduction in endothelial dysfunction are 50 and 100 mg/kg [11]. Our research shows that *p*-cymene exerts a dose-dependent analgesic effect (doses 5 to 50 mg/kg) in a carrageenan-induced hyperalgesia model [6].

Until now, we were primarily interested in investigations of the mechanism of anthelmintic action of EOAIs. We proved that carvacrol is a non-competitive antagonist of different types of nicotinic-acetyilcholine receptors (nAChR) of parasitic nematodes and that it most likely binds to the orthosteric site on the receptor [12]. The action of carvacrol on mammalian muscle-type nAChR is somewhat different. Our studies show that carvacrol acts on the presynaptic nAChR causing tetanic fade [12]. On the other hand, a single oral administration of carvacrol to rats disrupts motor coordination on Rota-rod test with an ED_50_ value of 421.6 mg/kg. However, no obvious signs of brain or nervous system depression were seen. The animals still had normal reflexes, could walk normally on a flat surface after being pinched on the tail, and all responded normally to external stimuli like approaching or touching [12]. Long-term antiparasitic, or any other therapeutic administration of carvacrol and *p*-cymene would involve the issue of their potential side effects and toxicity for the host. For this reason, we were interested to examine the impact of subacute administration of these two terpenes and the consequences on the general health condition, integration of the rat CNS, i.e., motor coordination and balance. We also examined the movement of wild-type free-living nematode *Caenorhabditis elegans* after subacute exposure to carvacrol and *p*-cymene.

The goal of our research was to examine the safety of repeated administration of carvacrol and *p*-cymene in doses with proven pharmacodynamic effects (and higher) in the two species of experimental animals. We were interested in examining whether repeated administration of carvacrol and *p*-cymene results in their cumulative effect and whether the phenolic structure of carvacrol, in contrast to *p*-cymene, causes higher toxicity. It can be assumed with great certainty that carvacrol and *p*-cymene in proven doses will be applied repeatedly and for a prolonged period of time (for antiparasitic, analgesic, antiinflammatory and other indications), so this study provides data on the safety of their application. On the other hand, our aim was to compare the results obtained in the study on the rat model with the results on the experimental model free living nematode *C. elegans*.

## 2. Results

### 2.1. Determination of the Median Lethal Dose (LD_50_) of Carvacrol and p-Cymene in Rats

At the very beginning of the investigation, we determined the LD_50_ of carvacrol and *p*-cymene in rats and the LC_50_ of these two monoterpenoids for *C. elegans*. The calculated LD_50_ value of carvacrol was 790.15 ± 1.15 mg/kg. However, unlike carvacrol, it was not possible to calculate the LD_50_ value of *p*-cymene because even the maximum possible volume of *p*-cymene that was administrated, does not cause deaths. So, we can only state that the LD_50_ of *p*-cymene is above 3000 mg/kg.

### 2.2. Determination of the Median Lethal Concentration (LC_50_) of p-Cymene and Carvacrol in C. elegans

**Figure 1:**
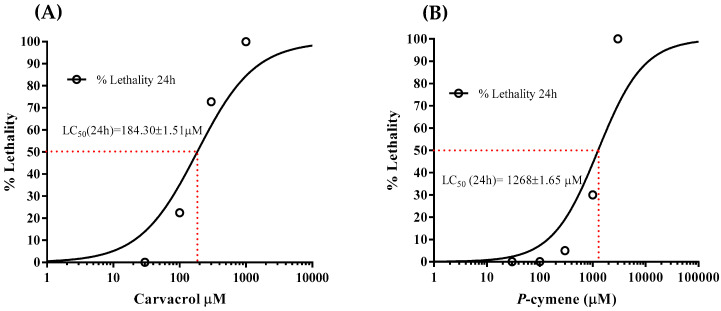
(**A**) Sigmoid curve of the relationship between the concentration of carvacrol and its lethal effect for adult *C. elegans*; (**B**) Sigmoid curve of the relationship between the concentration of *p*-cymene and its lethal effect for adult *C. elegans*.

The LC_50_ value of carvacrol for adult *C. elegans* was 184.13 ± 1.51 μM (Figure 1A). On the other hand, the LC_50_ value of *p*-cymene for adult *C. elegans* was 1268 ± 1.65 μM (Figure 1B).

### 2.3. Rota-Rod Performance

In the following third part of the research, we treated six groups of rats with increasing oral doses of carvacrol or *p*-cymene for 28 days, while the seventh group was an untreated control. Every day the rats were tested on the Rota-rod apparatus and the number of falls is recorded. During the entire observation period of 28 days, not a single fall was recorded in the rats treated with carvacrol or *p*-cymene. Furthermore, the righting reflex was completely preserved and movement on a flat surface was normal. No signs of CNS disorders, motor coordination problems, or other clinical symptoms were recorded.

### 2.4. Investigation of the Influence of Carvacrol and p-Cymene on the Motility of Adult C. elegans

In the examination of the subacute effects of carvacrol on adult *C. elegans*, it was shown that concentrations of 1, 10, and 100 μM do not affect the number of adults. The number increased every day and at the end of the trial it was 20 to 78% higher than at the beginning. However, the highest concentration of carvacrol tested (300 μM), caused a reduction in the number of adults by almost 50% (Table 1). Increasing concentrations of *p*-cymene of 30, 100 and 300 μM did not significantly affect the number of adults which increased by 60, 81, 3.5 and 116% after three days of exposure. However, the highest tested concentration of *p*-cymene of 1000 μM reduced the number of adults by more than 50%. In the control group, an increase in the number of adults by approximately 60% was recorded (Table 2).

**Figure 2:**
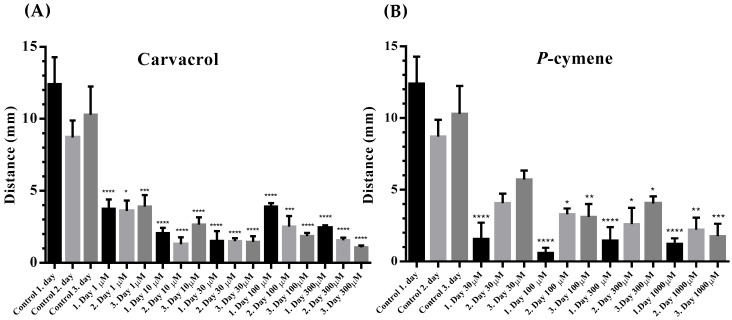
(**A**) Distance traveled during the three-day activity measurement of *C. elegans* (mm) in the presence of increasing concentrations of carvacrol (*n* = 3). Statistically significant difference compared to the control finding, every **** all means: ****—*p* < 0.0001; *—*p* = 0.0112; ***—*p* = 0.0008; ***—*p* = 0.0005. (**B**) Distance traveled during the three-day activity measurement of *C. elegans* (mm) in the presence of increasing concentrations of *p*-cymene (*n* = 3). Statistically significant difference compared to the control finding, every **** all means: ****—*p* < 0.0001; *—*p* = 0.0468; **—*p* = 0.021; *—*p* = 0.0150; *—*p* = 0.0141; **—*p* = 0.0076; ***—*p* = 0.0002.

All tested concentrations of carvacrol significantly reduced the distance of movement compared to control (Figure 2A). Similar to carvacrol, *p-*cymene caused a decrease in the movement distance of *C. elegans* in all applied concentrations (Figure 2B).

**Figure 3:**
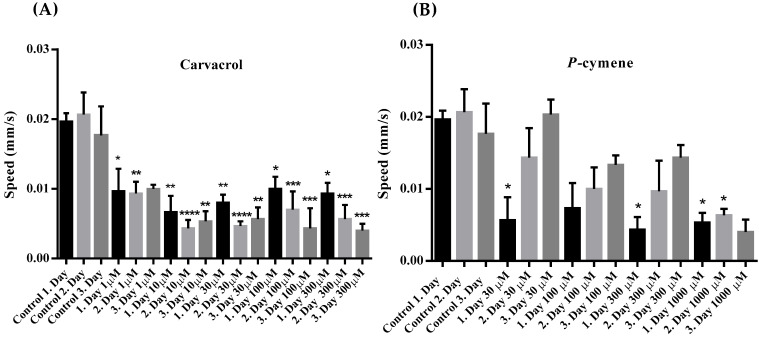
(**A**) Average movement speed of adult *C. elegans* measured during three days in the presence of increasing concentrations of carvacrol. Statistically significant difference compared to the control: *—*p* = 0.033; **—*p* = 0.0084; **—*p* = 0.0059; ****—*p* < 0.0001; **—*p* = 0.0013; **—*p* = 0.0028; ****—*p* < 0.0001; **—*p* = 0.0059; *—*p* = 0.0462; ***—*p* = 0.0006; ***—*p* = 0.0009; *—*p* = 0.0239; ***—*p* = 0.0001; ***—*p* = 0.0006. (**B**) Average movement speed of adult *C. elegans* measured during three days in the presence of increasing concentrations of *p*-cymene. Statistically significant difference compared to the control: *—*p* = 0.0422; *—*p* = 0.0178; *—*p* = 0.0342; *—*p* = 0.0342.

All tested concentrations of carvacrol significantly reduced the average speed of *C. elegans* movement in the 1st, 2nd and 3rd day of observation (Figure 3A). At the highest concentrations of carvacrol (100 and 300 μM), a decrease in movement speed was recorded from the 1st to the 3rd day of testing. It is interesting that in the presence of the *p*-cymene, the speed increased from the first to the third day, which was not observed in the control. This could be considered some kind of worm adaptation (Figure 3B).

**Figure 4:**
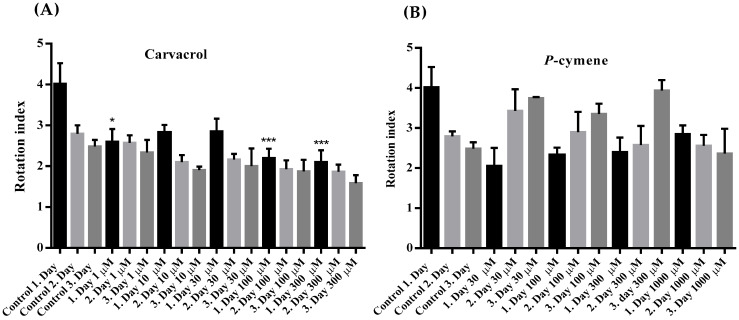
(**A**) Rotation index, measured during three-day exposure of adult *C. elegans* to increasing concentrations of carvacrol: *—*p* = 0.0279; ***—*p* = 0.0008; ***—*p* = 0.0004. (**B**) Rotation index, measured during three-day exposure of adult *C. elegans* to increasing concentrations of *p*-cymene.

**Figure 5:**
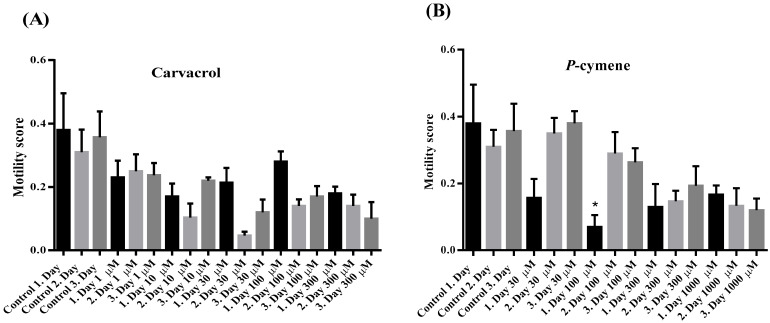
(**A**) Motility score of *C. elegans* in the presence of increasing concentrations of carvacrol and (**B**) *p*-cymene, *—*p* = 0.0364.

The rotation index of *C. elegans* was reduced under the influence of all applied concentrations of carvacrol compared to the control, but the reduction was significant only after the first 24 h of exposure to 1, 100 and 300 μM of carvacrol (Figure 4A). Otherwise, in the control and all other treated *C. elegans*, the rotation index increased insignificantly from the 1st to the 3rd day of exposure to carvacrol. Unlike carvacrol, *p*-cymene caused a non-significant increase in the rotation index in *C. elegans*, except at the highest concentration tested (Figure 4B). The motility score of *C. elegans* was non-significantly reduced at all tested concentrations of carvacrol and *p*-cymene (Figure 5A,B).

## 3. Discussion

Based on the results of the acute toxicity test, the LD_50_ of carvacrol was 790 mg/kg. Only one LD_50_ value for carvacrol has been found in the literature, at 810 mg/kg [13], which is in agreement with our findings. On the other hand, we were unable to determine the LD_50_ value of *p*-cymene because even the maximum volume that was applicable did not cause death in rats. This is the reason why we concluded that the LD_50_ of *p*-cymene in rats is higher than 3000 mg/kg. Data from the European Chemicals Agency (ECHA) show that the LD_50_ value of *p*-cymene determined by the Acute Toxic Class Method is 4750 mg/kg [14]. Obviously, this data is also in agreement with our results and indicate that *p*-cymene in comparison with carvacrol is far less acutely toxic for rats. Similarly to the results obtained in rats, carvacrol exhibited higher acute toxicity than *p*-cymene for *C. elegans*. The LC_50_ value of carvacrol obtained in this study corresponded to our previously published results [15]. However, the LC_50_ value of carvacrol obtained by Fuentes et al. [16] is much higher and was 1.10 ± 0.07 mM. The reason may be the medium in which the examination was carried out. We tested *C. elegans* in NGM medium, while Fuentes et al. [16] used a liquid medium. We emphasize that *E. coli*, which *C. elegans* feeds on, was present in both media, so the NGM medium probably provides better feeding efficiency (and thus the introduction of carvacrol by pharyngeal pumping), as well transcuticular penetration of carvacrol. Data on the LC_50_ value of p-cymene in *C. elegans* are not available in the literature. However, Lei et al. [17] state that concentration of *p*-cymene of 750 μM causes worm mortality of 16 ± 1%. Our results indicate that *p*-cymene exhibits lower acute toxicity than carvacrol, both for rats and for *C. elegans*. These two models exhibit a high degree of agreement that can be exploited in toxicological studies.

Doses of carvacrol and *p*-cymene of 50, 100 and 200 mg/kg administered orally for 28 days did not cause rats to fall off the rotating rod. The righting reflex was completely preserved and the rats moved normally on a flat surface. It is obvious that the tested doses applied for 28 days do not lead to disturbances in the integration of rats’ nervous system, nor do they affect the peripheral nervous system function. We have previously demonstrated that carvacrol exhibited a time and dose-dependent effect on the Rota-rod performances of rats, with a high value of the ED_50_ = 421.6 mg/kg. Furthermore, carvacrol acts on the presynaptic nACh receptor on the neuromuscular junction and produces tetanic fades in isolated rat diaphragm contractions [12]. Nevertheless, the ED_50_ value of carvacrol was cumulatively reached after two days of administration, but without the previously described effect on the Rota-rod test. For *p*-cymene, there are available data on mice test. Single intraperitoneal administration of *p*-cymene in doses of 25, 50, and 100 mg/kg did not induce changes in motor performance, since the animals remained on the rotating rod after 3, 30, 60, and 120 min [9].

Administration of a mixture of oregano oil (containing 36 to 80% carvacrol, other ingredients are not specified) and olive oil for 14 days in the diet of CRL Sprague Dawley CD^®^ IGS rats at a concentration of 1.25, 2.5, and 5.0% had no negative health effects [18]. In the subacute toxicity test of thyme oil, doses of 100, 250 and 500 mg/kg/day were administered for 28 days. Based on the analysis of the data obtained, the no-observed-adverse-effect level (NOAEL) is greater than 250 mg/kg/day. The main constituent of *Thymus vulgaris* essential oil was thymol (46.47%) and other constituents were γ-terpinene (20.27%), *p*-cymene (15.80%), α-terpinene (2.84%) and β-myrcene (1.91%) [19].

Our results are in agreement with published studies of prolonged toxicity of essential plant oils containing carvacrol and *p*-cymene. It is obvious that the mentioned essential oils as well as carvacrol and *p*-cymene, their constituents, do not show significant unwanted and toxic effects after prolonged use in experimental animals. It is obvious that carvacrol and *p*-cymene do not exert a cumulative toxic effect in rats. This point of view is supported by the fact that a single administration of high doses of carvacrol and *p*-cymene caused disturbances of the nervous system functions and clinical symptoms of toxicity. The absence of cumulative effects of carvacrol and *p*-cymene can be explained by their rapid biotransformation and elimination. Carvacrol is known to be rapidly metabolized and excreted in rats within the first 24 h of administration. Its excretion after 24 h is very limited, and the molecule is found unchanged [20]. After intragastric administration of *p*-cymene 100 mg/kg to rats, urinary metabolite excretion was nearly complete within 48 h, amounting to 60–80% of the dose [21].

Exposure of *C. elegans* to carvacrol in concentrations from 1 to 100 μM did not affect its development. The development of *C. elegans* from egg to adult at a temperature of 20 °C takes about 3 days [22]. So, we could expect an increase in the number of adults from the beginning to the end of our study. However, the highest tested concentration of carvacrol of 300 μM caused a 50% reduction in the number of adults, indicating that carvacrol interferes with the development of *C. elegans*. On the other hand, *p*-cymene reduced the number of adults by 50% only at a concentration of 1000 μM. Obviously, carvacrol exerts an effect on the development of *C. elegans* in a three times lower concentration than *p*-cymene.

The length of the traveled distance in the control worms did not differ during the three days of exposure. For 5 min of observation, the average distance covered on the first, second, and third day was 12.407 ± 1.874, 8.707 ± 1.117, 10.297 ± 1.946 mm. However, even the lowest tested concentration of carvacrol (1 μM) significantly reduced the length of the traveled distance to 3.750 ± 0.66, 3.610 ± 0.712 and 3.910 ± 0.800 mm. The other applied concentrations of carvacrol also caused a significant reduction in the distance, but at the same time, only at the two highest concentrations tested, 100 and 300 μM, the distance decreased from day 1 to day 3 (3.900 ± 0.240, 2.520 ± 0.736, 1.853± 0.225 mm and 2.433 ± 0.175, 1.570 ± 0.180, 1.080 ± 0.132 mm) (Figure 2A). The presence of *p*-cymene in all tested concentrations shortened the movement distance of *C. elegans* (Figure 2B). However, this reduction was highly significant only after the first day of exposure to all tested concentrations, and by the third day in all worms the distance increased (but without reaching the control level), which could indicate some kind of adaptation on the presence and effect of *p-*cymene.

A similar effect of carvacrol was recorded on the movement speed of *C. elegans*. All applied concentrations significantly decreased the speed compared to the control, but it is interesting that only the two highest concentrations also decreased the speed from the 1st to the 3rd day of observation (Figure 3A). Unlike carvacrol, *p*-cymene significantly reduced movement speed only at the highest tested concentration. Also, the speed was reduced on the first day after exposure to lower concentrations, but it was observed that on the 2nd and 3rd day, despite the presence of *p*-cymene, the speed of movement increased. This could be interpreted as some kind of adaptation of the worms to the presence of *p*-cymene. The observed effect of carvacrol on the length of the distance and the speed of movement can be explained by its proven inhibitory effect on the nicotinic acetylcholine receptor of nematodes [12,15]. This inhibition affects the speed of movement, and the distance covered. On the other hand, it is obvious that *p*-cymene exerts a weaker effect on these two observed parameters of *C. elegans* motility. It is important that *p*-cymene did not exhibit any inhibitory effect on contractions of the neuromuscular preparation of *A. suum* even at a concentration up to 1 mM (Supplementary Materials). We can assume that *p*-cymene affects *C. elegans* movement by a different mechanism compared to carvacrol.

The values of the average motility score and the rotation index are considered together. Carvacrol reduced the rotational index on the first day of observation but significantly in concentrations of 100 and 300 μM. At the same time, the motility score was also insignificantly reduced at all tested concentrations. On the other hand, the rotation index in worms exposed to *p*-cymene increased from the 1st to the 3rd day and was even higher than the control value, except at the highest tested concentration where it decreased until the third day. The motility score was lower than the control at all tested concentrations of *p*-cymen, but it also increased from the 1st to the 3rd day, except for the highest tested concentration. Rotation index is particularly useful for distinguishing between worms that are inactive and those that are merely rotating in place, as observed in certain roller strains. In the context of a paralysis assay where worms exhibit a low motility index, two scenarios arise: a low motility index with a low rotation index value typically indicates that the worms are motionless, which is observed as an effect of carvacrol; a low motility index with a high rotation index suggests that the worms are engaged in rolling movements, which is noted with *p*-cymene. This type of specific movement that occurs in some *C. elegans* mutants is a consequence of damage to the cuticle [23] or a maneuver for reorientation.

## 4. Materials and Methods

### 4.1. Animals

#### 4.1.1. Rats

White male albino Wistar rats (Mmab:W, Strain Report—Rat Genome Database), weighing 160–230 g were housed under standard conditions for laboratory animals in groups of five with controlled 12-h light/dark cycle, temperature of 21–24 °C, and ‘‘ad libitum” access to standard diet and water. All procedures in the study conformed to EEC Directive 86/609 and were approved by the Ethic Committee of the Faculty of Veterinary Medicine University of Belgrade and by Decision of the Veterinary Administration, Ministry of Agriculture, Forestry and Water Management, number: 7492 2023 14841 002 001 323 022, from 1 September 2024. At the end of the experiments all the rats were humanely euthanized by the overdose of pentobarbitone in accordance with the Home Office Code of Practice [24].

#### 4.1.2. *C. elegans*

*C. elegans*, wild type (N2 Bristol) was obtained from the Caenorhabditis Genetics Center [25]. Worms were cultivated and adults were separated for testing as we previously explained in Stojković et al. [15].

### 4.2. Chemicals and Method of Administration

Sigma Chemical Co. (St. Louis, MO, USA) supplied *p*-cymene (purity ≥ 97%) and carvacrol (purity ≥ 97%). Both were dissolved in peanut oil and administered to rats orally by gavage in a volume of 0.1 mL/100 g of body weight.

### 4.3. Procedures

#### 4.3.1. Determination of the Median Lethal Dose (LD_50_) of Carvacrol and *p*-Cymene in Rats

Fifty-four male rats were split into nine groups with six rats in each group. After a period of adaptation, the rats were given increasing doses of carvacrol through oral gavage: 400, 600, 800, and 1000 mg per kilogram of body weight, or *p*-cymene: 500, 1000, 2000, and 3000 mg per kilogram. Following the treatment, the rats were observed for 24 h to check for any deaths, and the results were used to find out the LD_50_ values for both carvacrol and *p*-cymene.

#### 4.3.2. Determination of the Median Lethal Concentration (LC_50_) of *p*-Cymene and Carvacrol in *C. elegans*

To find out the LC_50_ of *p*-cymene and carvacrol, adult nematodes were placed in Petri dishes 3 cm in diameter. Each dish had 2.5 mL of NGM substrate mixed with different amounts of *p*-cymene (30, 100, 300, 1000, and 3000 μM) or carvacrol (30, 100, 300, and 1000 μM). The number of worms in each 20-microliter sample was between 20 and 37. Each concentration was tested on three Petri dishes. The determination of LC_50_ is described in Stojković et al. [15].

#### 4.3.3. Rota-Rod Performance

The Rota-rod test is used to check how well rodents can keep their balance and coordination [26]. This test evaluates how long a rat can stay balanced on a rotating rod (El Unit, Belgrade, Serbia). Before starting the tests, the animals were trained for three days so they could stay on the rod for 180 s while it spins at a steady speed of 8 rpm. The rats were divided into seven groups with six rats in each group. Each group was given carvacrol or *p*-cymene dissolved in peanut oil at doses of 50, 100, and 200 mg/kg for 28 days. Every day, they did a 3-min test on the Rota-rod machine, and the number of times they fell off was recorded. The righting reflex and the test of moving on a flat surface were used to check if the doses of *p*-cymene and carvacrol had any noticeable effects on the rats’ central nervous system. All the tests were done following the methods described in Trailović et al. [27].

#### 4.3.4. Investigation of the Influence of Carvacrol and *p*-Cymene on the Motility of Adult *C. elegans*

*C. elegans*, N2 wild type was obtained from the Caenorhabditis Genetics Center [25]. The effect of *p*-cymene and carvacrol on the motility of adult *C. elegans* was investigated by using the Microtracker SMART 8 device (PHYLUMTECH S.A., Sunchales, Santa Fe, Argentina). In the Petri dishes (diameter 3 cm) filled with 2.5 mL of NGM substrate and increasing concentrations of *p*-cymene (30, 100, 300 and 1000 μM) or carvacrol (1, 10, 30, 100 and 300 µM) the suspension (20 μL) of adult *C. elegans* was added. Data collection was performed once a day, after 24, 48 and 72 h of exposure during a five-minute interval. The plates were subjected to “tapping” stimulation before each measurement. The following *C. elegans* motility parameters were monitored and analyzed: number of adults, movement speed, distance traveled, rotation index and motility score.

### 4.4. Statistics

All values are expressed as mean ± standard error of the mean (mean ± SE). One-way ANOVA test was used to determine the difference between groups in *C. elegans* study, followed by Tukey’s multiple comparisons test with 95% CI. Differences were considered significant at *p* < 0.05. Furthermore, the Brown-Forsythe test was used to check the equality of group variances (homogeneity of variance). Nonlinear regression with sigmoidal dose-response (variable slope) was used to examine dose/concentration-response relationship and determination of the Median Lethal Dose (LD_50_) as well as the Median Lethal Concentration (LC_50_) of carvacrol and *p*-cymene with 95% Confidence Intervals.

All data was analyzed using GraphPad Prism version 6.00 for Windows (GraphPad Software, La Jolla, CA, USA).

## 5. Conclusions

The results obtained definitely indicate that *p*-cymene exhibits lower acute toxicity than carvacrol, both for rats and *C. elegans* which may be due to its phenolic structure. These two models exhibit a high degree of agreement that can be exploited in toxicological studies. Tested doses of carvacrol and *p*-cymene administered for 28 days did not exert any effect on the CNS of rats or cause any clinical disorders. The absence of cumulative effects is probably due to rapid biotransformation and elimination in rats. This is supported by the fact that a cumulative effect was recorded in *C. elegans*. Carvacrol exhibited negative effects on *C. elegans* reproduction, distance traveled, movement speed, and rotational index at lower concentrations than *p*-cymene, indicating higher toxicity. On the other hand, although less toxic to *C. elegans*, *p*-cymene exhibited a specific effect on worm motility, with more rolling, which is characteristic of mutants with damaged cuticle or a maneuver for reorientation. In any case, this effect of *p*-cymene should be further investigated because it indicates a specific action.

## Figures and Tables

**Table 1:** The number of *C. elegans* adults during a 3-day exposure to increasing concentrations of carvacrol (*n* = 3).

	Control	Carvacrol 1 μM	Carvacrol 10 μM	Carvacrol 30 μM	Carvacrol 100 μM	Carvacrol 300 μM
Day 1	20.00 ± 4.16	14.33 ± 7.84	19.00 ± 11.79	20.00 ± 8.33	8.33 ± 6.33	10.00 ± 2.65
Day 2	27.67 ± 6.38	18.33 ± 10.34	21.67 ± 7.22	24.00 ± 8.00	11.67 ± 4.67	8.00 ± 1.73
Day 3	32.33 ± 2.03	25.66 ± 3.38	25.67 ± 3.38	24.67 ± 7.88	14.33 ± 5.49	5.33 ± 1.33

**Table 2:** The number of *C. elegans* adults during a 3-day exposure to increasing concentrations of *p*-cymene (*n* = 3).

	Control	*p*-Cymene 30 μM	*p-*Cymene 100 μM	*p*-Cymene 300 μM	*p*-Cymene 1000 μM
Day 1	20.00 ± 4.16	22.00 ± 10.26	29.00 ± 10.44	12.00 ± 2.52	32.67 ± 2.40
Day 2	27.67 ± 6.38	25.33 ± 8.51	28.00 ± 4.93	11.00 ± 2.65	27.67 ± 11.15
Day 3	32.33 ± 2.03	40.00 ± 4.00	30.33 ± 2.90	26.00 ± 0.58	16.00 ± 3.21

## Data Availability

The data that support the findings of this study are incorporated into the article.

## Supplementary Materials

The following supporting information can be downloaded at: https://www.mdpi.com/article/10.3390/molecules31071119/s1. Contractions of the isolated neuromuscular preparation of *Ascaris suum* in the presence of *p*-cymene.
