# Supplementary material for: Comparative Study of the Effects of Carvacrol and p-Cymene on the Motor Activity of Rats and Movement of Caenorhabditis elegans
Source: Molecules. 2026 Mar 28;31(7):1119. doi: 10.3390/molecules31071119 (PMC13074280; doi:10.3390/molecules31071119)
Supplement: Supplementary file 1 [file molecules-31-01119-s001.zip › molecules-4198384-supplementary.pdf]

***Ascaris suum*** neuromuscle preparation contractions, original recording.

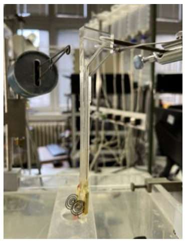

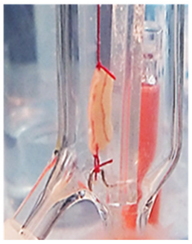

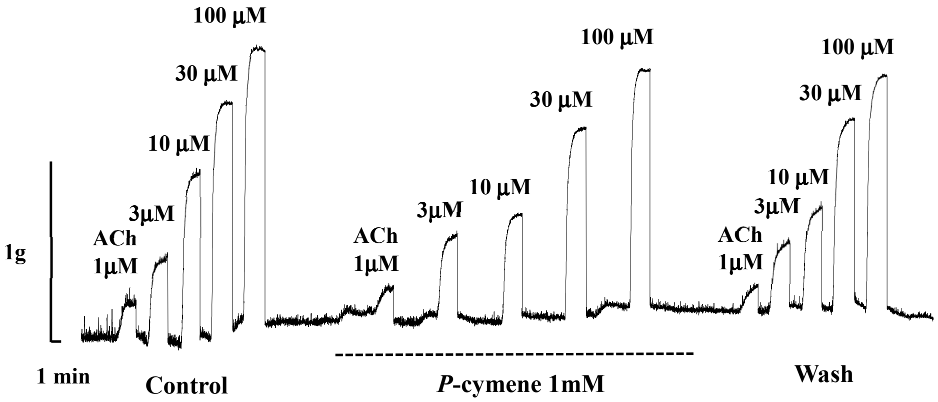


| **Control** (x ±SE) | | ***P*-cymene 1mM** (x ±SE) | | **Wash** (x ±SE) | |
|---|---|---|---|---|---|
| $EC_{50}$ | $E_{max}$ | $EC_{50}$ | $E_{max}$ | $EC_{50}$ | $E_{max}$ |
| 6.55±1.19µM | 1.54±0.07g | 7.11±1.16µM | 1.49±0.06g | 7.15±1.20µM | 1.39±0.09g |
